# Comparative Effects of Umbilical Cord- and Menstrual Blood-Derived MSCs in Repairing Acute Lung Injury

**DOI:** 10.1155/2018/7873625

**Published:** 2018-06-27

**Authors:** Haitao Ren, Qiang Zhang, Jinfu Wang, Ruolang Pan

**Affiliations:** ^1^Department of Burns and Wound Center, The Second Affiliated Hospital, School of Medicine, Zhejiang University, Hangzhou 310009, China; ^2^Key Laboratory of Cell-Based Drug and Applied Technology Development in Zhejiang Province, Hangzhou 311121, China; ^3^Institute for Cell-Based Drug Development of Zhejiang Province, S-Evans Biosciences, Hangzhou 311121, China; ^4^Institute of Cell and Development, College of Life Science, Zhejiang University, Hangzhou 310058, China

## Abstract

Mesenchymal stem cells (MSCs) can effectively relieve acute lung injury (ALI) in several *in vivo* models. However, the underlying mechanisms and optimal sources of MSCs are unclear. In the present study, we investigated the effects of umbilical cord- (UC-) and menstrual blood- (MB-) derived MSCs on ALI. MSCs were transplanted into a lipopolysaccharide-induced ALI mouse model, and the therapeutic effects were determined by histological, cellular, and biochemical analyses. Our results showed that both UCMSC and MBMSC transplantation inhibited the inflammatory response and promoted lung tissue repair. UCMSC treatment resulted in reduced damage and inflammation in the lung tissue and enhanced protection of lung function. Furthermore, we found that UCMSCs secreted higher levels of anti-inflammatory cytokines (interleukin-10 and keratinocyte growth factor) in ALI-related conditions, which may be due to the greater therapeutic capacity of UCMSCs compared with MBMSCs. These findings suggest that MSCs protected the lipopolysaccharide-induced ALI model by regulating inflammation, most likely via paracrine factors. Moreover, MSCs derived from the UC may be a promising alternative for ALI treatment.

## 1. Introduction

Mesenchymal stem cells (MSCs) are stromal cells that can differentiate into various cell types such as osteoblasts, chondrocytes, adipocytes, myocytes, and hepatocytes [1, 2]. In addition to their presence in the bone marrow, MSCs have been found in multiple tissues, including umbilical cord blood, adipose tissue, placenta, adult muscle, and even menstrual blood [3, 4]. Promising features such as multipotency, secretion of growth factors, and immunoregulatory properties make MSCs suitable candidates for cell-based therapies [5]. Numerous studies have demonstrated the beneficial effects of MSC-based therapy for various diseases in animal models and clinical trials, such as in liver fibrosis, cartilage regeneration, nerve injury, and wound healing [6–9].

Acute lung injury (ALI) is a major cause of acute respiratory failure and has a high mortality rate in critical care medicine [10]. Although ALI pathophysiology and treatments have been investigated in many studies, effective pharmacotherapies or therapeutic strategies are limited [11]. MSCs may be a promising alternative for treating lung diseases [12, 13], as increasing evidence supports the therapeutic effects of MSCs in pulmonary fibrosis, bronchopulmonary dysplasia, chronic obstructive pulmonary disease, and ALI. Recent studies have also shown that transplantation of MSCs from the bone marrow, umbilical cord (UC), menstrual blood (MB), and adipose tissue can attenuate lung injury and inflammatory responses [14–16]. Mechanistic studies revealed that MSCs can differentiate into lung tissue cells or exhibit paracrine functions [17–19]. For further application in ALI therapy, additional studies are needed to optimize several parameters of MSC therapy, such as cell sources, infusion routes, and doses.

In the present study, we compared the effects of UC- and MB-derived MSCs (UCMSCs and MBMSCs, resp.) on ALI using a lipopolysaccharide- (LPS-) induced mouse model. MSCs were intravenously transplanted into ALI animals, and their therapeutic effects were determined by histological, cellular, and biochemical analyses. We also preliminarily investigated the underlying mechanism by examining several cytokines secreted from these two types of MSCs.

## 2. Materials and Methods

### 2.1. Animals and Cells

Six- to eight-week-old imprinting control region (ICR) male mice were obtained from the Laboratory Animal Unit of Zhejiang Academy of Medical Sciences (Hangzhou, China). All animal experiments were performed in accordance with legal regulations and were approved by a local ethics committee. UCMSCs and MBMSCs were provided by S-Evans Biosciences (Hangzhou, China) and characterized as described previously [20]. Briefly, for UCMSC isolation, umbilical cord tissues (5–10 cm) were procured from healthy women during labor (*n* = 3) and cut into approximately 1 mm^3^ pieces for primary adherent culturing. MBMSCs were isolated from menstrual blood samples collected from healthy female donors (*n* = 3) with a menstruation cup (S-Evans Biosciences). Cells that reached 80–90% confluence was digested with 0.25% trypsin-EDTA (Gibco, Carlsbad, CA) for passaging. UCMSCs and MBMSCs of passage 3 were characterized by morphology, surface marker expression, and mesenchymal lineage differentiation (osteogenic, adipogenic, and chondrogenic differentiation), after which they were used for further experiments.

### 2.2. Experimental Models and Treatments

For the ALI model, ICR mice were treated with LPS as described previously [21], with some modifications. Briefly, animals were anesthetized with pentobarbital (60 mg/kg) intraperitoneally and then intratracheally injected with either 2 mg/kg LPS (*Escherichia coli* O55 : B5, Sigma-Aldrich, St. Louis, MO) dissolved in 100 *μ*L phosphate-buffered saline (PBS) or vehicle (PBS). After 6 h, 1 × 10^6^ UCMSCs or MBMSCs in 100 *μ*L PBS were transplanted intravenously into ALI mice through the tail vein and were defined as UCMSC- or MBMSC-treated groups. The animals were sacrificed 72 h posttransplantation, and samples were collected for further analysis.

### 2.3. Histological Analysis

Lung tissues were fixed with 4% paraformaldehyde and embedded in paraffin. Next, 5 *μ*m tissue sections were deparaffinized and stained with hematoxylin and eosin. The extent of lung injury was assessed under a light microscope (Carl Zeiss, Oberkochen, Germany) and semiquantified by determining the lung injury score as described previously [22].

### 2.4. Wet/Dry Ratio Determination

Lung tissues were collected and weighed immediately to determine wet weight. The tissues were dried in an oven at 60°C for 48 h to determine dry weight. The wet/dry ratio was then calculated and defined as the *W*/*D* ratio [15].

### 2.5. Arterial Blood Gas Analysis

Arterial blood gas was analyzed as descried previously [23, 24]. Briefly, mice were anesthetized with pentobarbital (60 mg/kg) intraperitoneally. All animals breathed spontaneously during the experiment. Then, blood samples were obtained from the celiac artery by heparinized syringes and immediately analyzed for oxygen partial pressure and oxygen saturation (sO_2_) using an ABL700 blood gas analyzer (Radiometer, Copenhagen, Denmark). The oxygen partial pressure/fractional inspired oxygen (pO_2_/FiO_2_) ratio was also calculated.

### 2.6. Measurements of Bronchoalveolar Lavage Fluid (BALF)

Mice were sacrificed, and the lungs were washed twice with 1 mL PBS for BALF sample collection. BALF was centrifuged at 300 ×g for 10 min. Cell pellets were resuspended in 200 *μ*L PBS for total cell and neutrophil count using a Countstar IC1000 (Beijing, China). The supernatants were collected to detect total protein concentrations using a Bicinchoninic Acid (BCA) Protein Assay kit (Beyotime Biotechnology, Jiangsu, China), and myeloperoxidase (MPO) activity was determined using an MPO kit (Nanjing Jiancheng Technology, Ltd., Nanjing, China) according to the manufacturer's instructions.

### 2.7. Enzyme-Linked Immunosorbent Assay (ELISA) Analysis

The concentrations of interleukin- (IL-) 1*β* and tumor necrosis factor *α* (TNF*α*) in BALF and serum as well as IL-10 and keratinocyte growth factor (KGF) in lung tissues were measured using ELISA kits (RayBiotech Inc., Norcross, GA) according to the manufacturer's instructions.

### 2.8. Treatment of MSCs with BALF-S

BALF from ALI mice were collected and centrifuged as described above. The supernatants were then passed through a 0.22 *μ*m filter and defined as BALF-S. Next, 1 × 10^6^ UCMSCs or MBMSCs of passage 3 were cultured and treated with 5% (*v*/*v*) BALF-S for 12 h. The cells were further placed in serum-free medium for another 24 h. Cultured media were collected and passed through a 0.22 *μ*m filter. Secreted IL-10 and KGF in the filtrate were examined using ELISA kits (RayBiotech) according to the manufacturer's instructions. Media from normal cultured UCMSCs or MBMSCs were used as controls. Furthermore, cell viability was measured using a Cell Counting Kit-8 assay (Beyotime Biotechnology) according to the manufacturer's instructions.

### 2.9. Statistical Analysis

All experiments were conducted at six times. Data are presented as the mean ± standard deviation. Statistical evaluation of differences between the values was determined by independent multiple Student's *t*-tests. *P* values of less than 0.05 were considered statistically significant.

## 3. Results

### 3.1. MSC Transplantation Attenuated Lung Injury in LPS-Induced ALI

**Figure 1:**
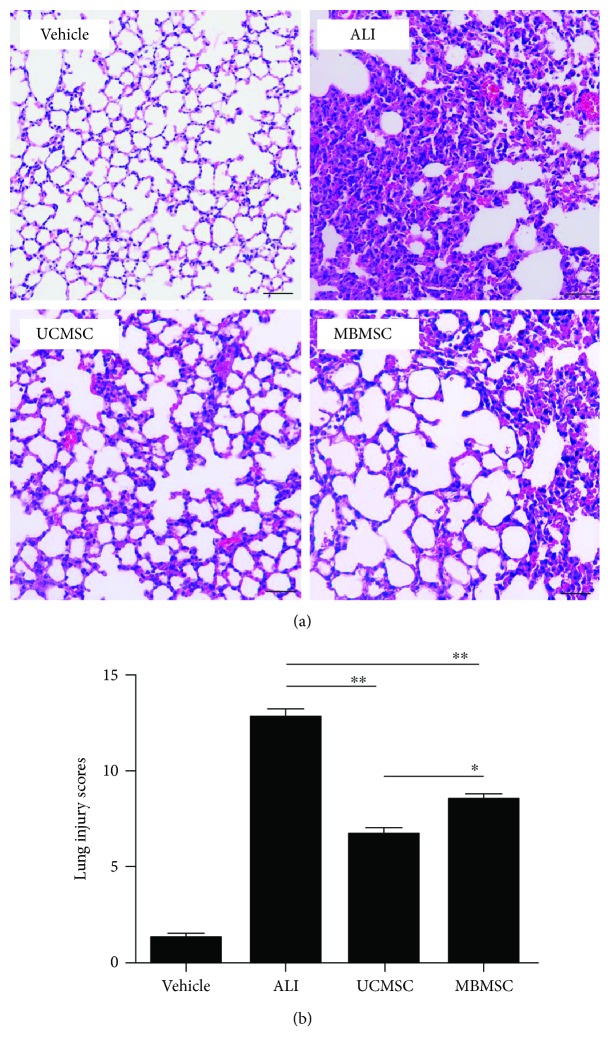
Assessment of histological changes in lung tissue. (a) HE-stained lung histological sections and representative lung histological changes. (b) Assessment of lung injury scores. The degree of lung injury was assessed from six sections, which conclude atelectasis, alveolar and interstitial inflammation, alveolar and interstitial hemorrhage, alveolar and interstitial edema, necrosis, and overdistension. Vehicle: normal mice with PBS injection; ALI: LPS-induced ALI model; UCMSC: ALI mice with UCMSC transplantation; MBMSC: ALI mice with MBMSC transplantation; HE: hematoxylin and eosin. Scale bars 50 *μ*m. ^∗^ *p* < 0.05, ^∗∗^ *p* < 0.01; *n* = 6.

UCMSCs and MBMSCs of passage 3 were characterized (Supplementary Figures 1 and 2) and transplanted intravenously into ALI mice. The therapeutic effects of MSCs on ALI were first evaluated by scoring hematoxylin and eosin-stained lung histological sections. The results showed that LPS-induced inflammatory infiltrates, interalveolar septal thickening, and other structural destruction were reduced by treatment with both UCMSCs and MBMSCs (Figure 1(a)). The degree of lung injury was further assessed by lung injury score evaluation based on atelectasis, alveolar and interstitial inflammation, alveolar and interstitial hemorrhage, alveolar and interstitial edema, necrosis, and overdistension. UCMSC administration resulted in a more significant reduction in lung injury compared with that in the MBMSC-treated group, as determined by lung injury score (6.7 ± 0.3 versus 8.5 ± 0.2, resp.; Figure 1(b)). These results suggest that MSCs can improve damaged lung tissue in ALI, with UCMSCs showing greater efficiency.

### 3.2. MSC Treatment Improved Pulmonary Function

**Figure 2:**
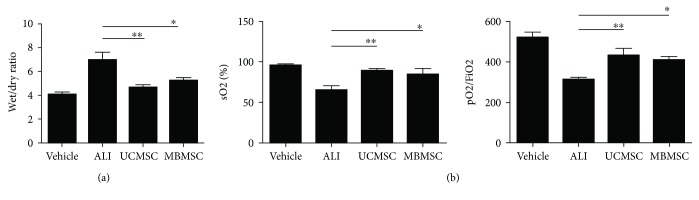
Indicator detections of pulmonary function. (a) Wet/dry ratio analysis. (b) The percentage of sO2 and the pO2/FiO2 ratio. Vehicle: normal mice with PBS injection; ALI: LPS-induced ALI model; UCMSC: ALI mice with UCMSC transplantation; MBMSC: ALI mice with MBMSC transplantation. ^∗^ *p* < 0.05, ^∗∗^ *p* < 0.01; *n* = 6.

To evaluate the role of MSCs in the repair of pulmonary function [15, 23], *W*/*D* ratios and arterial blood gases were measured. We found that ALI mice treated with UCMSCs and MBMSCs showed significantly lower *W*/*D* ratios (Figure 2(a)) of 4.7 and 5.3, respectively, versus 7.0 in ALI animals. This result indicates that treatment with either type of MSCs can decrease the degree of LPS-induced lung edema. Arterial blood gas analysis (Figure 2(b)) showed that LPS decreased the sO_2_ percentage (65.0%) and pO_2_/FiO_2_ ratio (314) compared with that of the vehicle group (95.5% and 519, resp.), whereas UCMSC and MBMSC treatments resulted in increased levels of both parameters, suggesting improvements in lung function recovery. Moreover, these results revealed that UCMSCs and MBMSCs have similar effects on lung function protection.

### 3.3. MSC Administration Reduced the Degree of Changes in BALF

**Figure 3:**
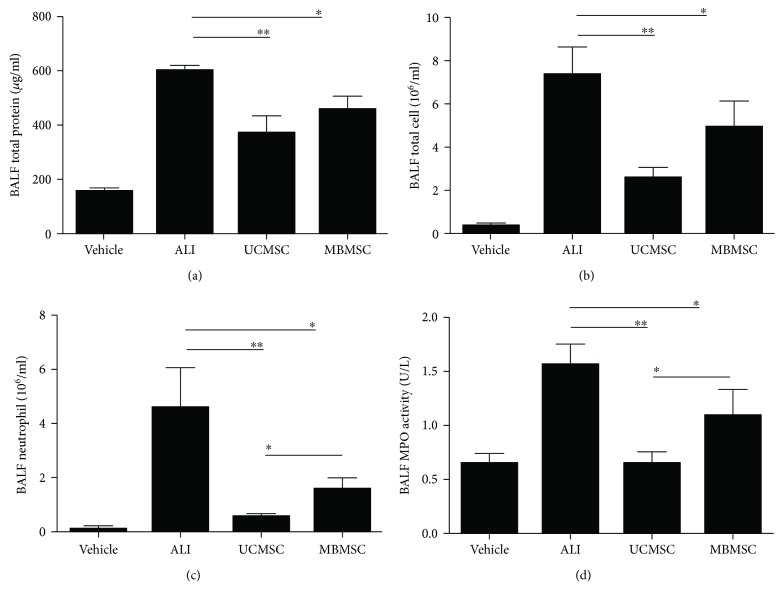
Detection of cell numbers and protein levels in BALF. (a) The concentrations of total protein in BALF. (b) Total cell counts in BALF. (c) Neutrophil counts in BALF. (d) Evaluation of MPO activity in BALF. Vehicle: normal mice with PBS injection; ALI: LPS-induced ALI model; UCMSC: ALI mice with UCMSC transplantation; MBMSC: ALI mice with MBMSC transplantation. ^∗^ *p* < 0.05, ^∗∗^ *p* < 0.01; *n* = 6.

To further analyze lung damage and inflammation, cellular counts and protein concentrations in BALF were examined. Total protein levels and cell numbers in BALF increased in LPS-induced ALI mice but decreased in UCMSC- and MBMSC-treated groups (Figures 3(a) and 3(b)). The numbers of neutrophils in BALF were significantly elevated by LPS induction but decreased in both MSC-treated groups. Moreover, we found that UCMSC transplantation resulted in a greater reduction in neutrophil numbers compared with MBMSCs (0.6 ± 0.08) × 10^6^/mL versus (1.6 ± 0.41) × 10^6^/mL, respectively (Figure 3(c)). This was further supported by MPO activity measurements, which gave values of 0.7 ± 0.11 U/L for the UCMSC group and 1.1 ± 0.22 U/L for the MBMSC group (Figure 3(d)). These results suggest not only that both types of MSCs do improve lung damage in ALI but also that UCMSCs can reduce cellular infiltration in an efficient manner.

### 3.4. MSCs Regulate the Expression of Inflammatory Cytokines

**Figure 4:**
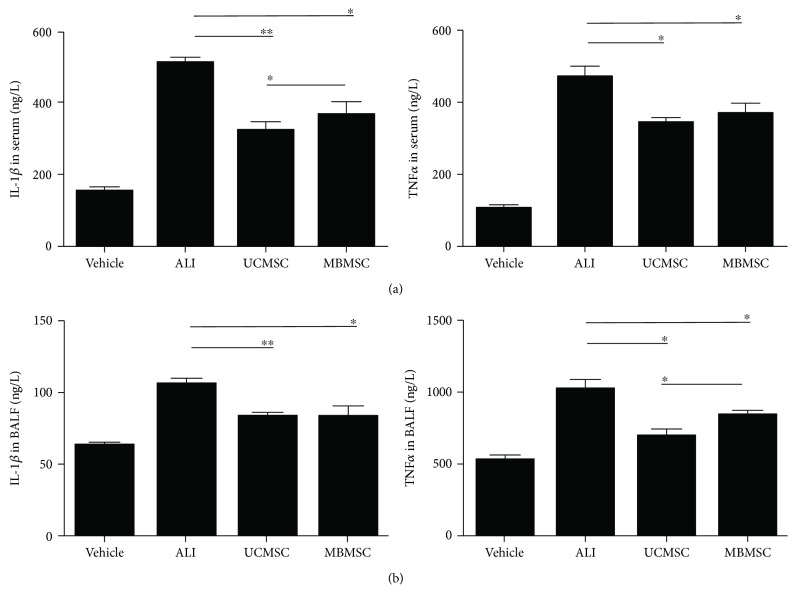
Analysis of inflammatory cytokines in serum and BALF. (a, b) The expression levels of IL-1*β* and TNF*α* in serum and BALF were detected by ELISA. Vehicle: normal mice with PBS injection; ALI: LPS-induced ALI model; UCMSC: ALI mice with UCMSC transplantation; MBMSC: ALI mice with MBMSC transplantation. ^∗^ *p* < 0.05, ^∗∗^ *p* < 0.01; *n* = 6.

To investigate inflammatory regulation by MSCs, we analyzed expression levels of the proinflammatory cytokines IL-1*β* and TNF*α* in serum (Figure 4(a)) and BALF (Figure 4(b)) via ELISA. The concentration of IL-1*β* and TNF*α* in serum and BALF was clearly elevated in the LPS-induced group and significantly reduced after MSC transplantation. The UCMSC-treated group showed a greater reduction in serum IL-1*β* (323 ± 23.9 ng/L) and BALF TNF*α* (692 ± 53.9 ng/L) levels compared with the MBMSC-treated group (368 ± 36.4 ng/L and 850 ± 25.4 ng/L, resp.). These results indicate that both types of MSCs decrease the expression of inflammatory cytokines.

### 3.5. MSC Treatment Upregulated the Expression of IL-10 and KGF in Lung Tissues

**Figure 5:**
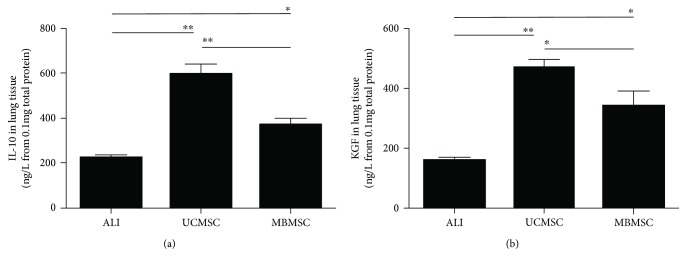
Examine the anti-inflammatory factor levels in lung tissues. (a, b) Expression levels of IL-10 and KGF in the lung tissues were detected by ELISA. ALI: LPS-induced ALI model; UCMSC: ALI mice with UCMSC transplantation; MBMSC: ALI mice with MBMSC transplantation. ^∗^ *p* < 0.05, ^∗∗^ *p* < 0.01; *n* = 6.

The expression of IL-10 (Figure 5(a)), a representative anti-inflammatory cytokine, was elevated both in MSC-treated groups and in the LPS-induced group. The results also showed that UCMSCs induced much higher levels of IL-10 in lung tissues compared with MBMSCs. Similarly, KGF, a potent mitogenic factor in alveolar epithelial cells, also showed increased expression in the MSC group, particularly in the UCMSC-treated group (Figure 5(b)). These findings suggest that MSCs attenuate lung injury and the inflammatory response by regulating the expression of several crucial factors.

### 3.6. BALF-S Stimulates IL-10 and KGF Secretion by MSCs

**Figure 6:**
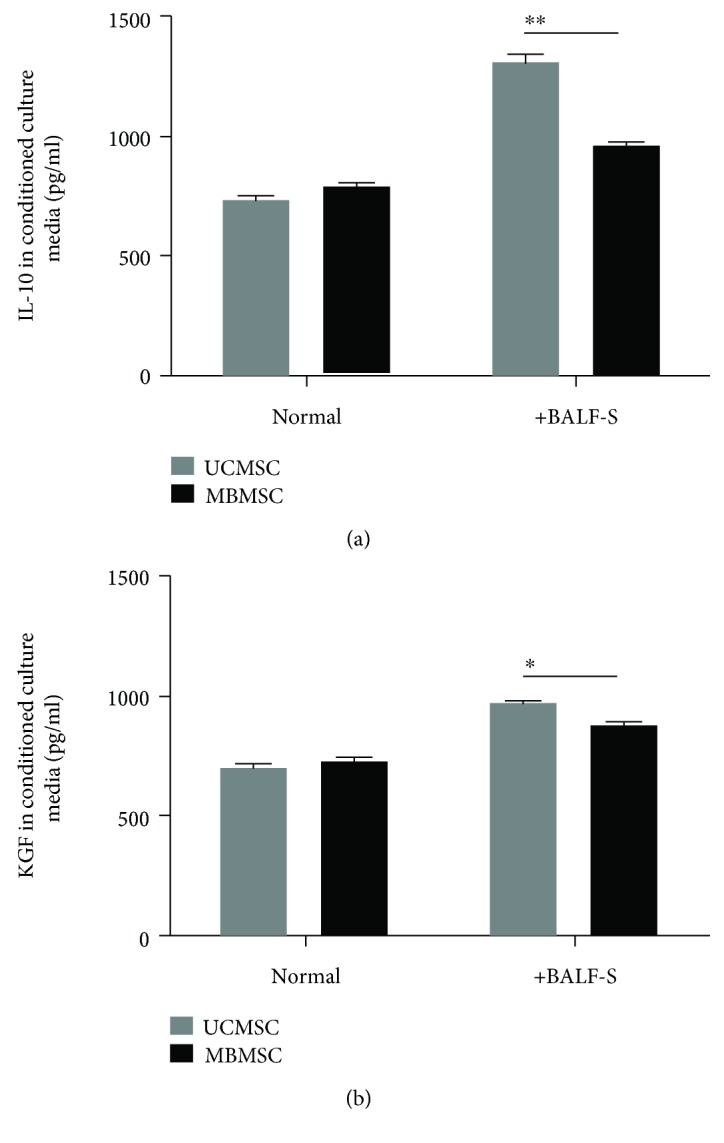
BALF-S-induced production of anti-inflammatory cytokines in cultured MSCs. (a, b) UCMSCs and MBMSCs were stimulated by 5% BALF-S (*v*/*v*), and the paracrine secretions of IL-10 and KGF in culture media were detected by ELISA. Normal: normal cultured groups; +BALF-S: 5% BALF-S-treated groups. ^∗^ *p* < 0.05, ^∗∗^ *p* < 0.01; *n* = 6.

We further investigated the secretion of soluble factors by MSCs which may contribute to the upregulation of IL-10 and KGF in lung tissues after MSC treatment. Paracrine factors IL-10 and KGF secreted by UCMSCs and MBMSCs after BALF-S stimulation were measured in culture media (Figure 6). The results showed that both types of MSCs secreted the two factors at comparable levels under normal culture conditions. After BALF-S treatment, expression levels of IL-10 and KGF increased to different extents and without any significant changes in cell viability (Supplementary Figure 3). BALF-S stimulation resulted in significantly enhanced secretion of IL-10 and KGF by UCMSCs (from 731 to 1316 pg/mL and 700 to 976 pg/mL, resp.) but not by MBMSCs. These results indicate that paracrine factors secreted by MSCs may partially contribute to the increased levels of IL-10 and KGF in MSC-treated lung tissues. Moreover, UCMSCs produced increased levels of cytokines or paracrine factors in response to the inflammatory condition, resulting in the more efficient in anti-inflammatory regulation observed in the UCMSC-treated group.

## 4. Discussion

The main findings of this study were that MSCs from both sources reduced lung injury and improved lung function in LPS-induced ALI mice to different extents, that MSCs may inhibit inflammatory responses by secreting anti-inflammatory cytokines to relieve lung injury, and that UCMSCs exhibited greater therapeutic effects than MBMSCs. This difference may be due to the increased secretion of anti-inflammatory cytokines by UCMSCs after transplantation in an inflammatory environment rather than differences in cell retention between MBMSCs and UCMSCs in injured lung tissue (Supplementary Figure 4). However, the underlying mechanism regarding whether secretion of other anti-inflammatory cytokines or exosomes by UCMSCs are involved requires further analysis.

Over the past decades, numerous preclinical studies on MSC-based therapies have been conducted due to the promising features of MSCs. Studies on lung disease therapies have focused on the ability of MSCs to secrete soluble factors, such as anti-inflammatory and cytokine growth factors, which can stabilize the alveolocapillary barrier, enhance alveolar fluid clearance, and decrease infection [25–28]. Clinical trials of lung disease therapies have also been conducted in recent years. In a double-blind randomized single-center trial, Zheng et al. [26] found that it is safe to inject human MSCs intravenously in 12 acute respiratory distress syndrome patients. In 2015, Wilson et al. [27] showed that intravenous administration of human MSCs was well tolerated in 9 patients with acute respiratory distress syndrome in a phase 1 clinical trial; based on these promising results, a phase 2 clinical study is currently underway.

Although the results are promising, the optimal dose, route of MSC administration, MSC sources, and precise mechanisms remain unclear. As a potential mechanism, the therapeutic effect of MSCs may be due to the secretion of soluble factors [28]. In ALI models, IL-10, prostaglandin E2, and KGF were shown to be secreted by MSCs to inhibit lung inflammation or protect against alveolar epithelium injury [29–32]. Our findings support the notion that MSCs secrete paracrine anti-inflammatory cytokines (IL-10 and KGF) to attenuate the inflammatory response and ameliorate lung injury. More importantly, we found that UCMSCs are more sensitive to inflammatory conditions and produced more cytokines or paracrine factors for lung repair in ALI. Although it is theoretically easy to obtain MBMSCs from monthly menstrual blood, we found that menstrual blood is prone to microbial contamination during the collection process (unpublished data). It was also found that MBMSCs have a weaker amplification capability compared with UCMSCs [20]. Taking these findings into consideration, UCMSCs appear to be more feasible for application in future ALI therapies. However, uncovering the precise mechanisms requires further investigation.

## 5. Conclusions

Our findings strongly support the use of UCMSCs and MBMSCs in ALI and other inflammatory lung disease therapies. Moreover, UCMSCs showed enhanced therapeutic effects compared with MBMSCs, indicating that UCMSCs are more promising for ALI treatment. Nevertheless, the specific mechanism underlying MSC-based ALI therapy requires further investigation.

## Data Availability

The datasets generated during and/or analyzed during the current study are available from the corresponding author on a reasonable request.

## Supplementary Materials

Supplementary Materials Supplementary Figure 1: characterization of UCMSC and MBMSC by morphological analysis and measurement of representative surface markers. Supplementary Figure 2: characterization of UCMSC and MBMSC by mesenchymal lineage differentiation. Supplementary Figure 3: cell viability assay for BALF-S-treated UCMSC and MBMSC. Supplementary Figure 4: detection of retained UCMSC and MBMSC in lung tissues at 72 h posttransplantation.
