# Supplementary material for: Comparative Effects of Umbilical Cord- and Menstrual Blood-Derived MSCs in Repairing Acute Lung Injury
Source: Stem Cells Int. 2018 Jun 27;2018:7873625. doi: 10.1155/2018/7873625 (PMC6040282; doi:10.1155/2018/7873625)
Supplement: Supplementary Materials — Supplementary Figure 1: characterization of UCMSC and MBMSC by morphological analysis and measurement of representative surface markers. Supplementary Figure 2: characterization of UCMSC and MBMSC by mesenchymal lineage differentiation. Supplementary Figure 3: cell viability assay for BALF-S-treated UCMSC and MBMSC. Supplementary Figure 4: detection of retained UCMSC and MBMSC in lung tissues at 72 h posttransplantation. [file 7873625.f1.pdf]

Supplementary Figure 1

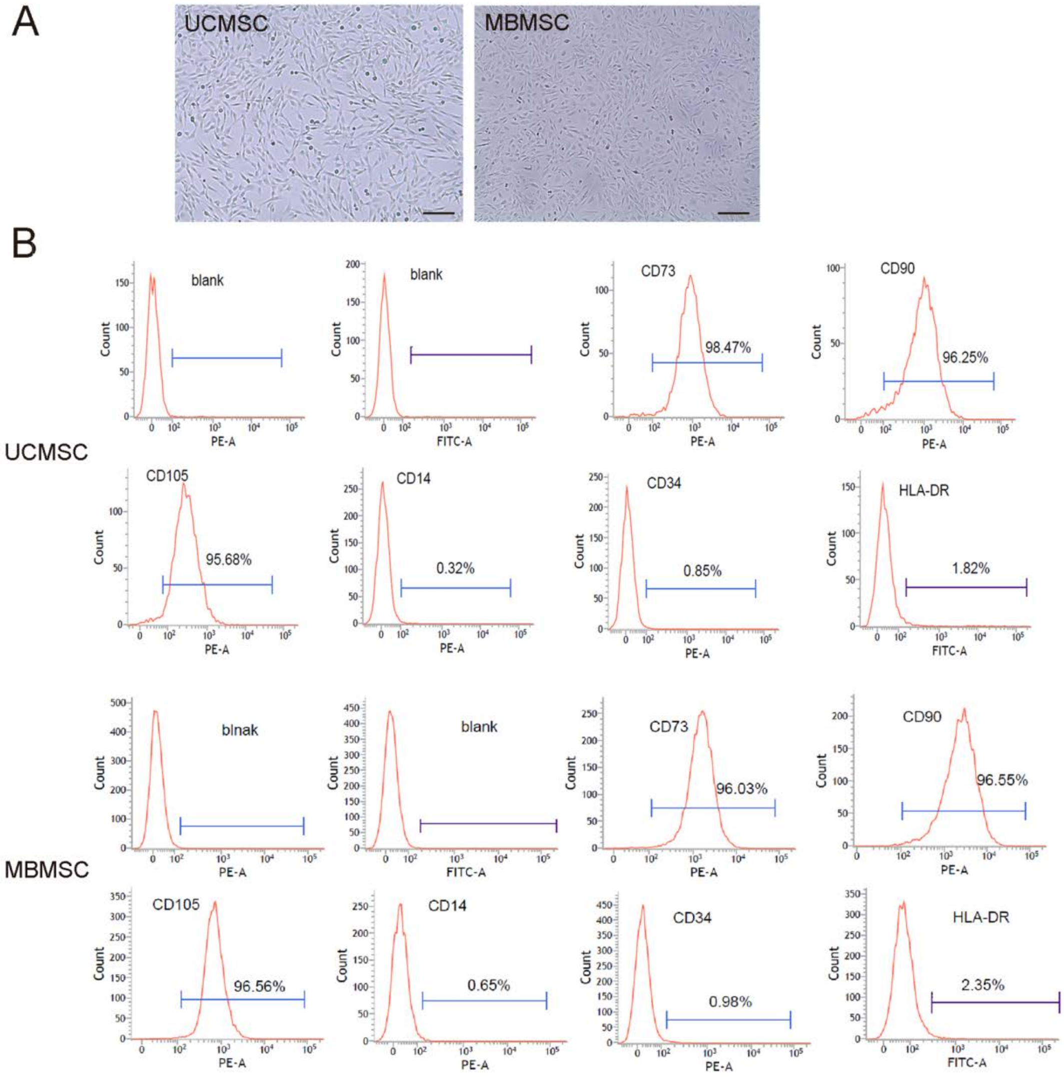


Supplementary Figure 1: Characterization of UCMSC and MBMSC. (A) Morphological analysis by microscopy. (B) Detection of representative surface markers by flow cytometer. Scale bars: 50μm.

Supplementary Figure 2

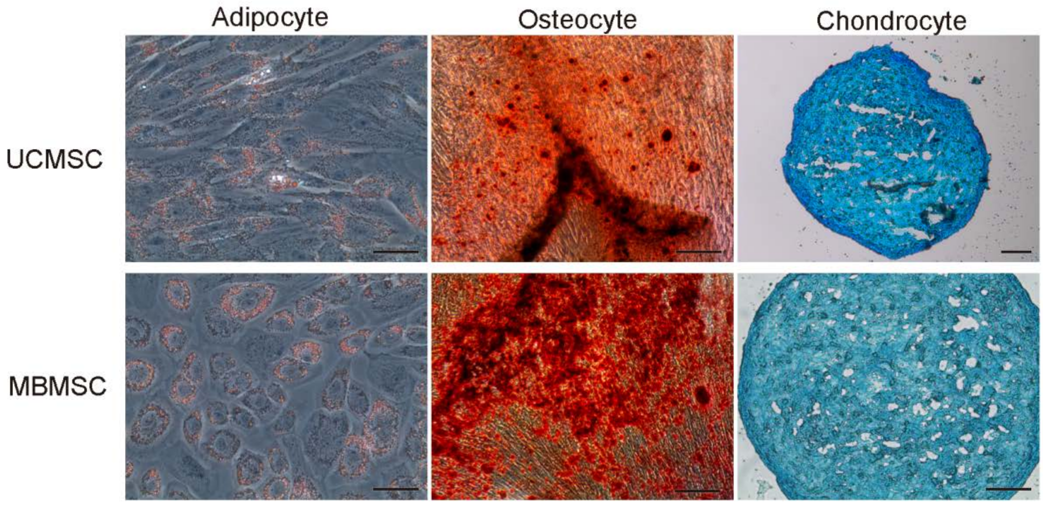


Supplementary Figure 2: Characterization of UCMSC and MBMSC by mesenchymal lineage differentiation. Adipocyte: adipogenic differentiation; Osteocyte: osteogenic differentiation; Chondrocyte: chondrogenic differentiation. Scale bars: 100μm.

Supplementary Figure 3

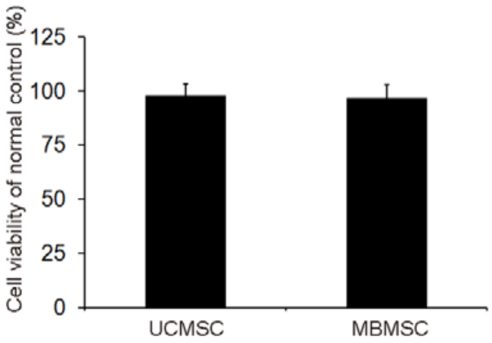


Supplementary Figure3: Cell viability assay. UCMSC and MBMSC represent groups of BALF-S-treated UCMSC and MBMSC.

Supplementary Figure 4

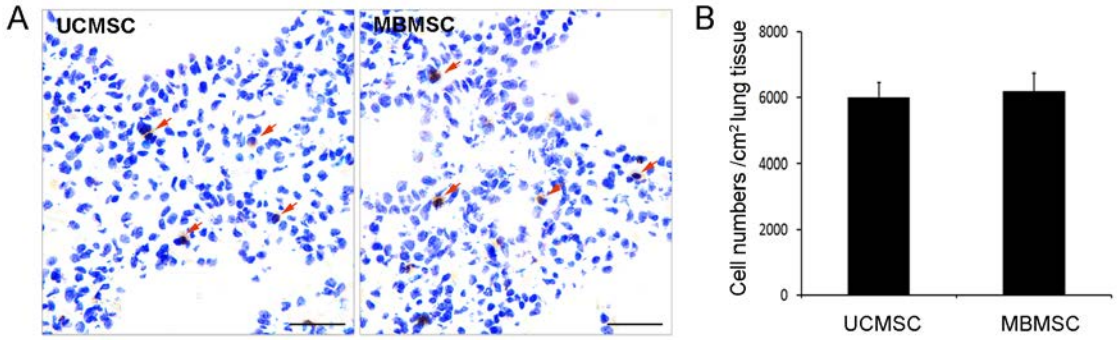


Supplementary Figure 4: Detection of retained UCMSC and MBMSC in lung tissues at 72 h post-transplantation. A, Immunostaining for human-specific nuclei antibody in lung tissues from MSC-treated ALI mice. Tissue sections were counterstained with hematoxylin. Arrows indicate human-specific nuclei antibody positive cells. Scale bars: 50μm; B, Cell numbers per $cm^2$ lung tissue were quantified from five random nonoverlapping fields of each sample.
